# Bis(di-2-pyridyl­amine-κ^2^ *N*,*N*′)bis­(thio­cyanato-κ*N*)nickel(II)

**DOI:** 10.1107/S1600536811050197

**Published:** 2011-11-30

**Authors:** Liliana Dobrzańska

**Affiliations:** a Department of Chemistry, Katholieke Universiteit Leuven, Celestijnenlaan 200F - bus 2404, B-3001 Heverlee, Belgium, and Department of Chemistry, University of Stellenbosch, Private Bag X1, Matieland, South Africa

## Abstract

The mononuclear neutral title complex, [Ni(NCS)_2_(C_10_H_9_N_3_)_2_], shows a *cis*-octa­hedral geometry around the Ni^II^ ion, formed by two chelating di-2-pyridyl­amine (Hdpa) ligands and two thio­cyanate anions. Both amine H atoms are involved in N—H⋯S hydrogen bonding, resulting in the formation of layers of inter­linked mol­ecules parallel to the *ab* plane, which are further held together by weak π–π inter­actions between adjacent complexes, involving one ring of each dipyridyl­amine unit [centroid–centroid distance = 3.777 (4) Å], forming a three-dimensional assembly.

## Related literature

For previous studies on mononuclear Ni^II^, Cu^II^ and Zn^II^ complexes with amine ligands, see: Wrzeszcz *et al.* (2002 ▶); Dobrzańska *et al.* (2000 ▶, 2001 ▶). For the spectroscopic properties of the title bulk material, see: Burbridge & Goodgame (1968 ▶). For crystallographic reports on *trans*-octa­hedral [Ni(chelating *N,N*-ligand)_2_(NCS)_2_] complexes, see: Wang *et al.* (2010 ▶); Karadag *et al.* (2004 ▶); Ghosh *et al.* (1997 ▶); for *cis*-, see: Zhang *et al.* (2008 ▶); Zhao *et al.* (2006 ▶); Moore & Squattrito (1999 ▶). For information about the configurations adopted by the Hdpa ligand, see: Chung *et al.* (2010 ▶). For a crystallo­graphic report on diazido­bis­(di-2-pyridyl­amine)­nickel(II) monohydrate, see: Villanueva *et al.* (2004 ▶).
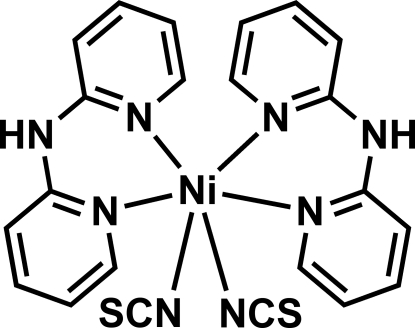


## Experimental

### 

#### Crystal data

[Ni(NCS)_2_(C_10_H_9_N_3_)_2_]
*M* *_r_* = 517.27
Monoclinic, 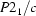

*a* = 8.605 (4) Å
*b* = 16.410 (9) Å
*c* = 16.556 (10) Å
β = 107.894 (8)°
*V* = 2225 (2) Å^3^
*Z* = 4
Mo *K*α radiation
μ = 1.09 mm^−1^
*T* = 100 K
0.24 × 0.18 × 0.04 mm

#### Data collection

Bruker APEX CCD area-detector diffractometer
Absorption correction: multi-scan (*SADABS*; Sheldrick, 1997 ▶) *T*_min_ = 0.780, *T*_max_ = 0.958
10729 measured reflections
4107 independent reflections
2815 reflections with *I* > 2σ(*I*)
*R*_int_ = 0.068

#### Refinement

*R*[*F* ^2^ > 2σ(*F* ^2^)] = 0.066
*wR*(*F* ^2^) = 0.161
*S* = 1.03
4107 reflections
304 parameters
H atoms treated by a mixture of independent and constrained refinement
Δρ_max_ = 0.77 e Å^−3^
Δρ_min_ = −0.84 e Å^−3^

### 

Data collection: *SMART* (Bruker, 2001 ▶); cell refinement: *SAINT* (Bruker, 2003 ▶); data reduction: *SAINT*; program(s) used to solve structure: *SHELXS97* (Sheldrick, 2008 ▶); program(s) used to refine structure: *SHELXL97* (Sheldrick, 2008 ▶); molecular graphics: *Mercury* (Macrae *et al.*, 2008 ▶); software used to prepare material for publication: *SHELXL97*.

## Supplementary Material

Crystal structure: contains datablock(s) I, global. DOI: 10.1107/S1600536811050197/pk2364sup1.cif

Structure factors: contains datablock(s) I. DOI: 10.1107/S1600536811050197/pk2364Isup2.hkl

Additional supplementary materials:  crystallographic information; 3D view; checkCIF report

## Figures and Tables

**Table 1:** Hydrogen-bond geometry (Å, °)

*D*—H⋯*A*	*D*—H	H⋯*A*	*D*⋯*A*	*D*—H⋯*A*
N7—H7⋯S32^i^	0.88 (5)	2.53 (6)	3.373 (5)	161
N20—H20⋯S29^ii^	0.85 (5)	2.59 (5)	3.437 (5)	175

Symmetry codes: (i) 
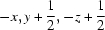
; (ii) 
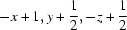
.

**Fig. 1.**
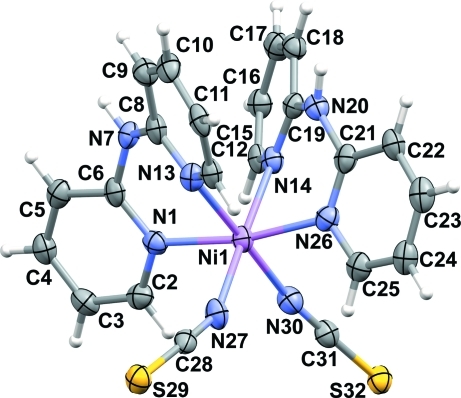
The molecular structure of the title compound, with the atom-numbering scheme. Displacement ellipsoids are drawn at the 50% probability level.

**Fig. 2.**
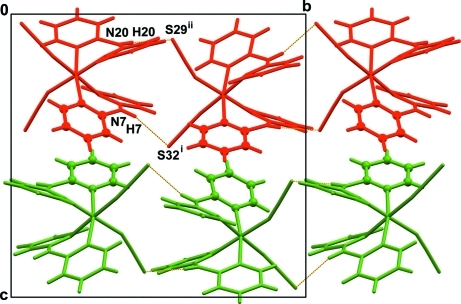
Capped sticks representation of the packing shown down the a axis; layers formed by N—H···S interactions (orange, symmetry codes: (i) -x, y + 1/2, -z + 1/2; (ii) -x + 1, y + 1/2, -z + 1/2) shown in red and green; π–π interactions highlighted by ball representation of the aromatic rings involved.

## supplementary crystallographic information

### Comment

As a continuation of our studies on NCS-bridged heteronuclear metal-organic complexes (Wrzeszcz *et al.*, 2002; Dobrzańska *et al.*, 2001; Dobrzańska *et al.*, 2000), based on mononuclear Ni^II^, Cu(II) and Zn(II) complexes with amine ligands, the title coordination compound was prepared. The spectroscopic properties of the title bulk material were reported earlier (Burbridge & Goodgame, 1968) but were not supported by crystallographic data. Nevertheless, there are quite a few crystallographic reports on [Ni(chelating *N,N*-ligand)_2_(NCS)_2_] complexes with *trans*- (Wang *et al.*, 2010; Karadag *et al.*, 2004; Ghosh *et al.*, 1997) or *cis*- (Zhang *et al.*, 2008; Zhao *et al.*, 2006; Moore & Squattrito, 1999) octahedral geometry. The complex crystallizes with one molecule in the asymmetric unit. It shows *cis*-octahedral arrangement of the ligands around Ni^II^, formed by two isothiocyanate anions and two chelating bidentate Hdpa ligands (Fig. 1). The latter adopt the most commonly encountered *anti-anti* configuration (Chung *et al.*, 2010). The pyridine rings in each Hdpa ligand are tilted with respect to one another with values of 33.4 (2)° for N7 and 31.0 (2)° for N20. A similar coordination environment has been reported for a related Ni^II^ complex with Hdpa and two azide ions instead of isothiocyanate ions (Villanueva *et al.*, 2004). The H amine atoms are involved in hydrogen bonding with the S atoms from the isothiocyanate ions of neighbouring molecules (Table 1), to form layers of interlinked molecules. These are further held together by weak π—π interactions between slightly tilted N1—C6 and C21—N26 rings with a centroid-centroid distance of 3.777 (4) Å (symmetry operation: *x*, 1/2 - *y*, 1/2 + *z*) to form a three-dimensional assembly (Fig. 2).

### Experimental

A methanolic solution (15 ml) of di-2-pyridylamine (171.2 mg, 1.0 mmol) was added dropwise to a methanolic solution (30 ml) of nickel thiocyanate (87.4 mg, 0.5 mmol). The reaction mixture was stirred for 15 minutes and left to evaporate in ambient air. After 3 weeks, crystals suitable for X-ray studies were obtained.

### Refinement

All phenyl H atoms were placed in calculated positions whereas amino H atoms were located in the Fourier difference map and their coordinates were freely refined. The *U*_iso_(H) values were set to 1.2*U*_eq_ of the carrying atom. The crystals diffract quite weakly at high angles which could be the reason the crystal structure has not been reported previously.

### Crystal data

**Table:**

[Ni(NCS)_2_(C_10_H_9_N_3_)_2_]	*F*(000) = 1064
*M**_r_* = 517.27	*D*_x_ = 1.544 Mg m^−^^3^
Monoclinic, *P*2_1_/*c*	Mo *K*α radiation, λ = 0.71073 Å
Hall symbol: -P 2ybc	Cell parameters from 2092 reflections
*a* = 8.605 (4) Å	θ = 2.5–24.8°
*b* = 16.410 (9) Å	µ = 1.09 mm^−^^1^
*c* = 16.556 (10) Å	*T* = 100 K
β = 107.894 (8)°	Plate, purple
*V* = 2225 (2) Å^3^	0.24 × 0.18 × 0.04 mm
*Z* = 4	

### Data collection

**Table:**

Bruker APEX CCD area-detector diffractometer	4107 independent reflections
Radiation source: fine-focus sealed tube	2815 reflections with *I* > 2σ(*I*)
graphite	*R*_int_ = 0.068
ω scans	θ_max_ = 25.6°, θ_min_ = 1.8°
Absorption correction: multi-scan (*SADABS*; Sheldrick, 1997)	*h* = −10→9
*T*_min_ = 0.780, *T*_max_ = 0.958	*k* = −12→19
10729 measured reflections	*l* = −17→19

### Refinement

**Table:**

Refinement on *F*^2^	Primary atom site location: structure-invariant direct methods
Least-squares matrix: full	Secondary atom site location: difference Fourier map
*R*[*F*^2^ > 2σ(*F*^2^)] = 0.066	Hydrogen site location: inferred from neighbouring sites
*wR*(*F*^2^) = 0.161	H atoms treated by a mixture of independent and constrained refinement
*S* = 1.03	*w* = 1/[σ^2^(*F*_o_^2^) + (0.076*P*)^2^ + 1.1451*P*] where *P* = (*F*_o_^2^ + 2*F*_c_^2^)/3
4107 reflections	(Δ/σ)_max_ < 0.001
304 parameters	Δρ_max_ = 0.77 e Å^−^^3^
0 restraints	Δρ_min_ = −0.84 e Å^−^^3^

### Special details

**Table:**

Geometry. All e.s.d.'s (except the e.s.d. in the dihedral angle between two l.s. planes) are estimated using the full covariance matrix. The cell e.s.d.'s are taken into account individually in the estimation of e.s.d.'s in distances, angles and torsion angles; correlations between e.s.d.'s in cell parameters are only used when they are defined by crystal symmetry. An approximate (isotropic) treatment of cell e.s.d.'s is used for estimating e.s.d.'s involving l.s. planes.
Refinement. Refinement of *F*^2^ against ALL reflections. The weighted *R*-factor *wR* and goodness of fit *S* are based on *F*^2^, conventional *R*-factors *R* are based on *F*, with *F* set to zero for negative *F*^2^. The threshold expression of *F*^2^ > σ(*F*^2^) is used only for calculating *R*-factors(gt) *etc*. and is not relevant to the choice of reflections for refinement. *R*-factors based on *F*^2^ are statistically about twice as large as those based on *F*, and *R*- factors based on ALL data will be even larger.

### Fractional atomic coordinates and isotropic or equivalent isotropic displacement parameters (Å^2^)

**Table:**

	*x*	*y*	*z*	*U*_iso_*/*U*_eq_	
Ni1	0.17658 (8)	0.22019 (4)	0.21548 (4)	0.0259 (2)	
N1	0.0773 (5)	0.2386 (3)	0.3150 (3)	0.0265 (10)	
C2	−0.0102 (6)	0.1787 (3)	0.3351 (3)	0.0309 (13)	
H2	−0.0192	0.1284	0.3057	0.037*	
C3	−0.0881 (7)	0.1865 (3)	0.3965 (4)	0.0321 (13)	
H3	−0.1484	0.1427	0.4095	0.038*	
C4	−0.0750 (7)	0.2606 (3)	0.4384 (3)	0.0313 (13)	
H4	−0.1284	0.2686	0.4802	0.038*	
C5	0.0151 (6)	0.3220 (3)	0.4194 (3)	0.0306 (13)	
H5	0.0255	0.3729	0.4479	0.037*	
C6	0.0914 (6)	0.3089 (3)	0.3575 (3)	0.0279 (12)	
N7	0.1791 (5)	0.3731 (3)	0.3382 (3)	0.0264 (10)	
H7	0.167 (7)	0.420 (3)	0.361 (4)	0.032*	
C8	0.3232 (7)	0.3684 (3)	0.3174 (3)	0.0290 (12)	
C9	0.4232 (7)	0.4366 (3)	0.3302 (3)	0.0332 (13)	
H9	0.3900	0.4866	0.3486	0.040*	
C10	0.5705 (7)	0.4300 (3)	0.3157 (3)	0.0328 (13)	
H10	0.6405	0.4760	0.3224	0.039*	
C11	0.6166 (7)	0.3557 (3)	0.2912 (3)	0.0318 (13)	
H11	0.7220	0.3486	0.2851	0.038*	
C12	0.5081 (7)	0.2924 (3)	0.2758 (3)	0.0296 (12)	
H12	0.5393	0.2418	0.2576	0.035*	
N13	0.3598 (5)	0.2989 (3)	0.2854 (3)	0.0269 (10)	
N14	0.0418 (5)	0.3193 (3)	0.1498 (3)	0.0251 (10)	
C15	−0.1120 (6)	0.3317 (3)	0.1526 (3)	0.0263 (12)	
H15	−0.1666	0.2881	0.1703	0.032*	
C16	−0.1916 (7)	0.4037 (3)	0.1314 (3)	0.0300 (12)	
H16	−0.3006	0.4096	0.1328	0.036*	
C17	−0.1126 (7)	0.4684 (4)	0.1075 (3)	0.0351 (13)	
H17	−0.1638	0.5202	0.0954	0.042*	
C18	0.0408 (7)	0.4562 (3)	0.1018 (3)	0.0310 (13)	
H18	0.0984	0.4994	0.0855	0.037*	
C19	0.1103 (6)	0.3797 (3)	0.1202 (3)	0.0248 (11)	
N20	0.2599 (5)	0.3658 (3)	0.1073 (3)	0.0265 (10)	
H20	0.306 (7)	0.410 (3)	0.100 (4)	0.032*	
C21	0.3201 (7)	0.2927 (3)	0.0869 (3)	0.0296 (13)	
C22	0.4206 (7)	0.2955 (3)	0.0350 (3)	0.0324 (13)	
H22	0.4455	0.3460	0.0137	0.039*	
C23	0.4827 (7)	0.2240 (4)	0.0153 (4)	0.0355 (14)	
H23	0.5566	0.2245	−0.0172	0.043*	
C24	0.4367 (7)	0.1514 (4)	0.0430 (3)	0.0332 (13)	
H24	0.4733	0.1009	0.0275	0.040*	
C25	0.3384 (6)	0.1539 (3)	0.0926 (3)	0.0306 (13)	
H25	0.3067	0.1037	0.1115	0.037*	
N26	0.2823 (5)	0.2237 (3)	0.1172 (3)	0.0285 (10)	
N27	0.3197 (6)	0.1248 (3)	0.2766 (3)	0.0332 (11)	
C28	0.4180 (7)	0.0920 (3)	0.3306 (3)	0.0276 (12)	
S29	0.55527 (18)	0.04778 (9)	0.41017 (9)	0.0335 (4)	
N30	−0.0026 (6)	0.1428 (3)	0.1499 (3)	0.0317 (11)	
C31	−0.0852 (7)	0.0975 (3)	0.1021 (4)	0.0282 (12)	
S32	−0.20188 (17)	0.03490 (8)	0.03303 (9)	0.0308 (3)	

### Atomic displacement parameters (Å^2^)

**Table:**

	*U*^11^	*U*^22^	*U*^33^	*U*^12^	*U*^13^	*U*^23^
Ni1	0.0245 (4)	0.0307 (4)	0.0212 (4)	0.0002 (3)	0.0048 (3)	0.0001 (3)
N1	0.025 (2)	0.028 (3)	0.025 (2)	0.0005 (19)	0.004 (2)	0.0037 (18)
C2	0.028 (3)	0.030 (3)	0.031 (3)	−0.002 (2)	0.003 (3)	0.000 (2)
C3	0.029 (3)	0.035 (3)	0.033 (3)	0.001 (2)	0.011 (3)	0.009 (2)
C4	0.028 (3)	0.042 (4)	0.024 (3)	0.004 (3)	0.008 (3)	0.003 (2)
C5	0.026 (3)	0.035 (3)	0.027 (3)	0.004 (2)	0.003 (2)	−0.003 (2)
C6	0.024 (3)	0.038 (3)	0.018 (3)	0.002 (2)	0.001 (2)	0.002 (2)
N7	0.024 (3)	0.031 (3)	0.022 (2)	−0.0015 (19)	0.005 (2)	−0.0050 (18)
C8	0.025 (3)	0.043 (3)	0.018 (3)	0.001 (2)	0.005 (2)	0.001 (2)
C9	0.033 (3)	0.038 (3)	0.028 (3)	−0.004 (3)	0.008 (3)	0.001 (2)
C10	0.028 (3)	0.043 (4)	0.025 (3)	−0.012 (3)	0.004 (3)	−0.003 (2)
C11	0.021 (3)	0.052 (4)	0.020 (3)	−0.005 (3)	0.002 (2)	0.005 (2)
C12	0.026 (3)	0.041 (3)	0.019 (3)	0.003 (2)	0.003 (2)	0.002 (2)
N13	0.020 (2)	0.034 (3)	0.025 (2)	0.0047 (18)	0.005 (2)	−0.0003 (19)
N14	0.021 (2)	0.032 (3)	0.021 (2)	−0.0015 (19)	0.0052 (19)	0.0000 (18)
C15	0.024 (3)	0.038 (3)	0.016 (3)	−0.001 (2)	0.004 (2)	−0.001 (2)
C16	0.024 (3)	0.038 (3)	0.026 (3)	0.002 (2)	0.004 (2)	0.000 (2)
C17	0.036 (3)	0.038 (3)	0.028 (3)	0.007 (3)	0.005 (3)	0.003 (2)
C18	0.032 (3)	0.034 (3)	0.025 (3)	−0.005 (2)	0.006 (2)	0.000 (2)
C19	0.022 (3)	0.028 (3)	0.023 (3)	0.001 (2)	0.005 (2)	−0.002 (2)
N20	0.021 (2)	0.033 (3)	0.025 (2)	−0.0040 (19)	0.006 (2)	0.0012 (19)
C21	0.024 (3)	0.037 (3)	0.023 (3)	−0.004 (2)	0.000 (2)	−0.005 (2)
C22	0.027 (3)	0.045 (4)	0.023 (3)	0.000 (3)	0.006 (2)	0.002 (2)
C23	0.021 (3)	0.057 (4)	0.028 (3)	−0.002 (3)	0.006 (2)	−0.003 (3)
C24	0.021 (3)	0.050 (4)	0.027 (3)	0.005 (3)	0.005 (2)	−0.004 (3)
C25	0.023 (3)	0.038 (3)	0.027 (3)	0.002 (2)	0.001 (2)	−0.001 (2)
N26	0.027 (2)	0.030 (3)	0.027 (2)	−0.001 (2)	0.006 (2)	−0.0029 (19)
N27	0.031 (3)	0.043 (3)	0.022 (3)	0.001 (2)	0.002 (2)	0.002 (2)
C28	0.032 (3)	0.030 (3)	0.025 (3)	−0.010 (2)	0.013 (3)	−0.004 (2)
S29	0.0313 (8)	0.0375 (9)	0.0283 (8)	0.0047 (6)	0.0040 (6)	0.0004 (6)
N30	0.029 (3)	0.037 (3)	0.029 (3)	−0.002 (2)	0.008 (2)	0.004 (2)
C31	0.023 (3)	0.035 (3)	0.029 (3)	0.000 (2)	0.012 (3)	0.002 (2)
S32	0.0280 (8)	0.0363 (8)	0.0259 (8)	−0.0042 (6)	0.0051 (6)	−0.0020 (6)

### Geometric parameters (Å, °)

**Table:**

Ni1—N30	2.035 (5)	C12—H12	0.9500
Ni1—N27	2.055 (5)	N14—C19	1.322 (6)
Ni1—N13	2.092 (4)	N14—C15	1.354 (6)
Ni1—N26	2.095 (4)	C15—C16	1.357 (7)
Ni1—N14	2.097 (4)	C15—H15	0.9500
Ni1—N1	2.097 (4)	C16—C17	1.382 (8)
N1—C6	1.337 (7)	C16—H16	0.9500
N1—C2	1.341 (7)	C17—C18	1.367 (8)
C2—C3	1.385 (7)	C17—H17	0.9500
C2—H2	0.9500	C18—C19	1.384 (7)
C3—C4	1.387 (8)	C18—H18	0.9500
C3—H3	0.9500	C19—N20	1.387 (7)
C4—C5	1.365 (8)	N20—C21	1.389 (7)
C4—H4	0.9500	N20—H20	0.85 (5)
C5—C6	1.392 (7)	C21—N26	1.318 (7)
C5—H5	0.9500	C21—C22	1.396 (8)
C6—N7	1.390 (7)	C22—C23	1.369 (8)
N7—C8	1.387 (7)	C22—H22	0.9500
N7—H7	0.88 (5)	C23—C24	1.378 (8)
C8—N13	1.335 (7)	C23—H23	0.9500
C8—C9	1.388 (8)	C24—C25	1.349 (7)
C9—C10	1.364 (8)	C24—H24	0.9500
C9—H9	0.9500	C25—N26	1.353 (7)
C10—C11	1.382 (8)	C25—H25	0.9500
C10—H10	0.9500	N27—C28	1.157 (7)
C11—C12	1.366 (7)	C28—S29	1.644 (6)
C11—H11	0.9500	N30—C31	1.157 (7)
C12—N13	1.337 (7)	C31—S32	1.632 (6)
			
N30—Ni1—N27	91.65 (19)	N13—C12—H12	118.7
N30—Ni1—N13	178.74 (17)	C11—C12—H12	118.7
N27—Ni1—N13	87.81 (18)	C8—N13—C12	117.7 (5)
N30—Ni1—N26	92.34 (18)	C8—N13—Ni1	121.2 (3)
N27—Ni1—N26	93.79 (18)	C12—N13—Ni1	118.0 (3)
N13—Ni1—N26	88.83 (17)	C19—N14—C15	116.9 (4)
N30—Ni1—N14	89.99 (18)	C19—N14—Ni1	122.3 (3)
N27—Ni1—N14	176.50 (17)	C15—N14—Ni1	119.3 (3)
N13—Ni1—N14	90.61 (17)	N14—C15—C16	122.9 (5)
N26—Ni1—N14	83.06 (17)	N14—C15—H15	118.5
N30—Ni1—N1	94.85 (18)	C16—C15—H15	118.5
N27—Ni1—N1	92.87 (17)	C15—C16—C17	119.3 (5)
N13—Ni1—N1	84.04 (17)	C15—C16—H16	120.3
N26—Ni1—N1	170.05 (17)	C17—C16—H16	120.3
N14—Ni1—N1	90.07 (16)	C18—C17—C16	118.6 (5)
C6—N1—C2	117.9 (5)	C18—C17—H17	120.7
C6—N1—Ni1	123.0 (3)	C16—C17—H17	120.7
C2—N1—Ni1	119.0 (4)	C17—C18—C19	118.6 (5)
N1—C2—C3	123.3 (5)	C17—C18—H18	120.7
N1—C2—H2	118.3	C19—C18—H18	120.7
C3—C2—H2	118.3	N14—C19—C18	123.4 (5)
C2—C3—C4	117.7 (5)	N14—C19—N20	118.3 (4)
C2—C3—H3	121.1	C18—C19—N20	118.3 (5)
C4—C3—H3	121.1	C19—N20—C21	127.8 (4)
C5—C4—C3	119.7 (5)	C19—N20—H20	112 (4)
C5—C4—H4	120.2	C21—N20—H20	118 (4)
C3—C4—H4	120.2	N26—C21—N20	119.7 (5)
C4—C5—C6	119.1 (5)	N26—C21—C22	122.3 (5)
C4—C5—H5	120.5	N20—C21—C22	117.9 (5)
C6—C5—H5	120.5	C23—C22—C21	118.6 (5)
N1—C6—N7	120.0 (5)	C23—C22—H22	120.7
N1—C6—C5	122.2 (5)	C21—C22—H22	120.7
N7—C6—C5	117.7 (5)	C22—C23—C24	119.3 (5)
C8—N7—C6	127.2 (5)	C22—C23—H23	120.4
C8—N7—H7	113 (4)	C24—C23—H23	120.4
C6—N7—H7	115 (4)	C25—C24—C23	118.4 (5)
N13—C8—N7	118.9 (5)	C25—C24—H24	120.8
N13—C8—C9	122.6 (5)	C23—C24—H24	120.8
N7—C8—C9	118.5 (5)	C24—C25—N26	123.8 (5)
C10—C9—C8	118.4 (5)	C24—C25—H25	118.1
C10—C9—H9	120.8	N26—C25—H25	118.1
C8—C9—H9	120.8	C21—N26—C25	117.4 (5)
C9—C10—C11	119.2 (5)	C21—N26—Ni1	122.4 (4)
C9—C10—H10	120.4	C25—N26—Ni1	119.4 (4)
C11—C10—H10	120.4	C28—N27—Ni1	157.0 (4)
C12—C11—C10	118.9 (5)	N27—C28—S29	177.5 (5)
C12—C11—H11	120.5	C31—N30—Ni1	166.4 (4)
C10—C11—H11	120.5	N30—C31—S32	178.8 (5)
N13—C12—C11	122.7 (5)		
			
N30—Ni1—N1—C6	−147.6 (4)	N30—Ni1—N14—C15	57.0 (4)
N27—Ni1—N1—C6	120.5 (4)	N13—Ni1—N14—C15	−121.9 (4)
N13—Ni1—N1—C6	33.1 (4)	N26—Ni1—N14—C15	149.3 (4)
N14—Ni1—N1—C6	−57.6 (4)	N1—Ni1—N14—C15	−37.9 (4)
N30—Ni1—N1—C2	29.7 (4)	C19—N14—C15—C16	−3.8 (7)
N27—Ni1—N1—C2	−62.2 (4)	Ni1—N14—C15—C16	162.6 (4)
N13—Ni1—N1—C2	−149.7 (4)	N14—C15—C16—C17	−1.6 (8)
N14—Ni1—N1—C2	119.7 (4)	C15—C16—C17—C18	3.5 (8)
C6—N1—C2—C3	1.0 (8)	C16—C17—C18—C19	−0.1 (8)
Ni1—N1—C2—C3	−176.4 (4)	C15—N14—C19—C18	7.5 (7)
N1—C2—C3—C4	0.5 (8)	Ni1—N14—C19—C18	−158.5 (4)
C2—C3—C4—C5	−1.2 (8)	C15—N14—C19—N20	−172.7 (4)
C3—C4—C5—C6	0.4 (8)	Ni1—N14—C19—N20	21.3 (6)
C2—N1—C6—N7	−179.1 (5)	C17—C18—C19—N14	−5.7 (8)
Ni1—N1—C6—N7	−1.9 (7)	C17—C18—C19—N20	174.5 (5)
C2—N1—C6—C5	−1.9 (8)	N14—C19—N20—C21	29.3 (8)
Ni1—N1—C6—C5	175.4 (4)	C18—C19—N20—C21	−150.9 (5)
C4—C5—C6—N1	1.2 (8)	C19—N20—C21—N26	−33.8 (8)
C4—C5—C6—N7	178.5 (5)	C19—N20—C21—C22	146.6 (5)
N1—C6—N7—C8	−38.8 (8)	N26—C21—C22—C23	−0.4 (8)
C5—C6—N7—C8	143.9 (5)	N20—C21—C22—C23	179.2 (5)
C6—N7—C8—N13	23.8 (8)	C21—C22—C23—C24	3.7 (8)
C6—N7—C8—C9	−156.7 (5)	C22—C23—C24—C25	−3.5 (8)
N13—C8—C9—C10	−5.3 (8)	C23—C24—C25—N26	−0.2 (8)
N7—C8—C9—C10	175.2 (5)	N20—C21—N26—C25	177.3 (5)
C8—C9—C10—C11	−1.7 (8)	C22—C21—N26—C25	−3.2 (8)
C9—C10—C11—C12	5.0 (8)	N20—C21—N26—Ni1	−13.4 (7)
C10—C11—C12—N13	−1.5 (8)	C22—C21—N26—Ni1	166.1 (4)
N7—C8—N13—C12	−171.8 (5)	C24—C25—N26—C21	3.5 (8)
C9—C8—N13—C12	8.7 (8)	C24—C25—N26—Ni1	−166.1 (4)
N7—C8—N13—Ni1	28.5 (6)	N30—Ni1—N26—C21	130.3 (4)
C9—C8—N13—Ni1	−151.0 (4)	N27—Ni1—N26—C21	−137.9 (4)
C11—C12—N13—C8	−5.2 (8)	N13—Ni1—N26—C21	−50.1 (4)
C11—C12—N13—Ni1	155.2 (4)	N14—Ni1—N26—C21	40.6 (4)
N27—Ni1—N13—C8	−139.8 (4)	N30—Ni1—N26—C25	−60.6 (4)
N26—Ni1—N13—C8	126.4 (4)	N27—Ni1—N26—C25	31.2 (4)
N14—Ni1—N13—C8	43.3 (4)	N13—Ni1—N26—C25	118.9 (4)
N1—Ni1—N13—C8	−46.7 (4)	N14—Ni1—N26—C25	−150.3 (4)
N27—Ni1—N13—C12	60.6 (4)	N30—Ni1—N27—C28	−152.9 (11)
N26—Ni1—N13—C12	−33.3 (4)	N13—Ni1—N27—C28	25.9 (11)
N14—Ni1—N13—C12	−116.3 (4)	N26—Ni1—N27—C28	114.6 (11)
N1—Ni1—N13—C12	153.7 (4)	N1—Ni1—N27—C28	−58.0 (11)
N30—Ni1—N14—C19	−137.4 (4)	N27—Ni1—N30—C31	−83 (2)
N13—Ni1—N14—C19	43.8 (4)	N26—Ni1—N30—C31	11 (2)
N26—Ni1—N14—C19	−45.0 (4)	N14—Ni1—N30—C31	94 (2)
N1—Ni1—N14—C19	127.8 (4)	N1—Ni1—N30—C31	−176 (2)

### Hydrogen-bond geometry (Å, °)

**Table:**

*D*—H···*A*	*D*—H	H···*A*	*D*···*A*	*D*—H···*A*
N7—H7···S32^i^	0.88 (5)	2.53 (6)	3.373 (5)	161
N20—H20···S29^ii^	0.85 (5)	2.59 (5)	3.437 (5)	175

Symmetry codes: (i) −*x*, *y*+1/2, −*z*+1/2; (ii) −*x*+1, *y*+1/2, −*z*+1/2.
